# Pickering emulsions stabilized by β-CD microcrystals: Construction and interfacial assembly mechanism

**DOI:** 10.3389/fnut.2023.1161232

**Published:** 2023-03-22

**Authors:** Xingran Kou, Xinping Zhang, Qinfei Ke, Qingran Meng

**Affiliations:** ^1^Collaborative Innovation Center of Fragrance Flavor and Cosmetics, School of Perfume and Aroma Technology (Shanghai Research Institute of Fragrance and Flavour Industry), Shanghai Institute of Technology, Shanghai, China; ^2^Key Laboratory of Textile Science and Technology, Ministry of Education, College of Textiles, Donghua University, Shanghai, China

**Keywords:** β-Cyclodextrin, microcrystals, Pickering emulsion, interfacial assembly, ginger oil

## Abstract

β-Cyclodextrin (β-CD) can combine with oil and other guest molecules to form amphiphilic inclusion complexes (ICs), which can be adsorbed on the oil–water interface to reduce the interfacial tension and stabilize Pickering emulsions. However, the subtle change of β-CD in the process of emulsion preparation is easily ignored. In this study, β-CD and ginger oil (GO) were used to prepare the Pickering emulsion by high-speed shearing homogenization without an exogenous emulsifier. The stability of the emulsion was characterized by microscopic observation, staining analysis, and creaming index (CI). Results showed that the flocculation of the obtained Pickering emulsion was serious, and the surface of the droplets was rough with lamellar particles. In order to elucidate the formation process of the layered particles, the GO/β-CD ICs were further prepared by ball milling method, and the X-ray diffraction (XRD), scanning electron microscope (SEM), Fourier transform infrared spectroscopy (FTIR), and interfacial tension analyses found that β-CD and GO first formed amphiphilic nanoscale small particles (ICs) through the host–guest interaction, and the formed small particles were further self-assembled into lamellar micron-scale amphiphilic ICs microcrystals. These amphiphilic ICs and microcrystals aggregated at the oil–water interface and finally formed the Pickering emulsion. In this study, by exploring the formation process and evolution of GO/β-CD self-assembly, the formation process and stabilization mechanism of the β-CD-stabilized GO Pickering emulsion were clarified preliminarily, with the aim of providing a theoretical basis for the development of high-performance CD-stabilized Pickering emulsions.

## 1. Introduction

Emulsions are heterogeneous solutions of mixtures in which one liquid is dispersed as drops in another (1). Generally, emulsifiers (surfactants, polymers, and solid particles) are usually needed to form a dynamic stable system. Emulsions stabilized by solid colloidal particles are called Pickering emulsions, and the solid particles here are called Pickering emulsifiers or Pickering particles (2). Such particles can be synthetic or natural but are different from traditional emulsifiers (1, 2). During the formation of Pickering emulsion, particles first gather at the oil–water interface, forming different types of emulsion, *i.e*., oil-in-water or water-in-oil emulsion (3). In food and pharmaceutical fields, the commonly used Pickering particles mainly include protein (zein, soy protein, and other protein particles), polysaccharides (starch, chitosan, celluloses, and other polysaccharide particles), cyclodextrins (CDs) and their derivatives, and other small particles. In other fields such as the daily chemical industry, leather industry, home textile industry, ecological environment field, and inorganic particles such as titanium dioxide particles, silica particles, and carbon nanotubes are commonly used as Pickering particles (4).

Cyclodextrins (CDs) are a kind of cyclic oligosaccharides with cyclic glucose as the basic structural unit, which is produced by the degradation of starch by cycloglycosyltransferases produced by various bacilli (5). CD family includes three major cyclic oligosaccharides, *i.e*., α-CD, β-CD, and γ-CD, which contain 6, 7, and 8 glucose units, respectively (5–9). CDs have a special cavity structure with lipophilic inside and hydrophilic outside, and such hydrophobic cavity can trap different kinds of hydrophobic guest molecules to form colloidal particles or inclusion complexes (ICs), which play an important role in drug delivery system (5, 10, 11). In addition, the aforementioned three CDs have matured production technologies and low prices and are also food additives specified in the USA, EU, China, and other countries and regions (6, 9, 12–16).

The inclusion complexes (ICs) formed by CDs and guest molecules can further self-assemble into microcrystals driven by non-specific electrostatic, π-π interaction, dispersion forces, hydrophobic, host–guest complexation, or “lock–key” specific binding (17–19). The formed ICs microcrystals are easily adsorbed to the oil–water interface, thereby stabilizing the Pickering emulsion. When other components such as surfactants or polymers are added to the IC systems, CD supramolecules can be further regulated to form colloidal particles such as micelles or vesicles (18, 20). However, when the concentration of CDs exceeds a certain level, the resulting vesicles can also be destroyed (21). Regarding the research status of ICs formed by β-CD, relevant research articles and review articles have been published by our research group (11, 21).

Ginger oil (GO) is a kind of oily liquid containing a variety of chemical components extracted from ginger rhizomes. GO contains a variety of active ingredients, such as gingerols, shogaol, and zingiberone (22, 23). Such numerous bioactive phytochemicals endow GO with a unique aroma. On the contrary, the existence of polyphenols and other antioxidant components enables GO to play antibacterial, antioxidative, and shelf-life-prolonging functions in the food and pharmaceutical fields (24). However, alkenes, ketones, aldehydes, alcohols, phenols, and other components contained in GO are very sensitive to temperature, light, processing technology, and storage conditions, which limit their application (23). In order to overcome these short planks, embedding, solubilization, and emulsification may be effective methods. While current research mainly focused on the encapsulation of GO, there is no report on CD-stabilized GO Pickering emulsion (25).

β-Cyclodextrin (β-CD) can combine with oil and other guest molecules to form amphiphilic inclusion complexes (ICs), which can be adsorbed on the oil–water interface to reduce the interfacial tension and stabilize Pickering emulsion. However, the subtle change of β-CD in the process of emulsion preparation is easily ignored. In this study, the GO was used as the guest to construct GO/β-CD ICs crystals to further form Pickering emulsion. The obtained emulsion was analyzed by optical and fluorescence microscopes, particle size analyzers, three-phase contact angles, X-ray diffraction (XRD), scanning electron microscope (SEM), and Fourier transform infrared spectroscopy (FTIR) to characterize the particle size, morphology, and ICs structure. Meanwhile, the influence of ICs morphology and host concentration on the host–guest self-assembly and morphology, particle size, microstructure, and interface properties of emulsion were also explored. Through the present study, it is hoped to clarify the self-assembly process of solid particles, discover the morphological evolution law of self-assembly, and reveal the principle of CDs crystallite-stabilized Pickering emulsion.

## 2. Materials and methods

### 2.1. Materials

β-CD (AR grade, >98.0%) and Nile blue A (>75%) were purchased from Shanghai Titan Chemical Co., Ltd. Nile red (RG grade) was obtained from Tokyo Chemical Industry Co., Ltd. GO was purchased from Lihe Flavor (Qingdao) Food Co., Ltd. Other chemicals used in the present study were of AR grade.

### 2.2. Samples preparation

#### 2.2.1. Preparation of β-CD solution

An aliquot of 5 g of β-CD was precisely weighed and placed into a 100-mL volumetric flask. An appropriate amount of deionized water was added, and the mixture was heated and stirred at 65°C for 20 min to fully dissolve the β-CD. Then, the solution was cooled to 45°C and diluted with deionized water (45°C) to volume to obtain the stock solution (5 wt%). Other β-CD aqueous solutions with different concentrations were obtained by diluting the stock solution with deionized water (45°C).

#### 2.2.2. Preparation of emulsion

An appropriate amount of β-CD aqueous solution was accurately measured into a beaker, and a certain amount of GO was added. The mixture was homogenized by a T10 basic Ultra-Turrax^®^ high-speed shear homogenizer (IKA, Germany) at 10,000 rpm for 2 min to obtain the GO/CD emulsion.

#### 2.2.3. Preparation of ICs

An aliquot of 19 mL of 5 wt% β-CD aqueous solution was accurately weighed and placed into a ball mill jar, and 1 mL of GO was added. The mixture was milled using an MM400 hybrid ball mill instrument (Retsh GmbH, Germany) at a vibration frequency of 30 Hz for 3 min. The grinding ball and grinding chamber are made of zirconia. The obtained sample was transferred into a tube and centrifuged at 8,000 rpm to obtain the precipitate. The precipitate was washed with distilled water at least three times to remove free β-CD and then dried at 60°C for 24 h to obtain a powder sample.

### 2.3. Analysis of emulsion

#### 2.3.1. Stability analysis

The prepared emulsion was transferred into a vial and stored statically at 25°C. The creaming index (CI) was calculated as follows:


$$\begin{array}{l} CI=\frac{H_{c}}{H_{t}}\times 100\% \end{array}$$

Of which, *H*_c_ represented the height of the emulsified layer; *H*_t_ represented the total height of the emulsion.

#### 2.3.2. Optical microscope observation analysis

An aliquot of 10 μL of the emulsion was transferred onto a clean glass slide, and a cover glass was put onto the emulsion drop gently. An Olympus VR BX 53 microscope (Olympus, Japan) with an Olympus DP74 and LC MICRO measurement software was used to observe and analyze the emulsion.

#### 2.3.3. Fluorescence microscope observation analysis

Before measurement, the aqueous phase of the emulsion was stained with Nile blue A, and the oily phase was stained with Nile red. Then, 10 μL of the stained emulsion was transferred onto a clean glass slide, and a cover glass was put onto the emulsion drop gently. An Olympus IX 53 fluorescence microscope (Olympus, Japan) with Olympus DP74 and cellSens standard analysis software was used to observe and analyze the emulsion.

#### 2.3.4. Particle size analysis

Based on the dynamic light scattering method, the particle size of the emulsion was determined using a Mastersizer 2000 laser particle size analyzer (Malvern Analytical Ltd., USA). In order to avoid the influence of multiple scattering during the test, the sample was pre-diluted 500–1000 times with deionized water in advance, and the refractive index of the dispersant and the dispersed phase was set to 1.33 and 1.59, respectively. During testing, the stirring speed was 2,400 rpm, and the average particle diameter of the emulsion was finally represented by the volume average diameter (*D*_[3,2]_). All samples were measured three times at room temperature, and finally, the average particle size was calculated.

### 2.4. Characterization of ICs

#### 2.4.1. SEM analysis

The prepared powder sample, as mentioned in Section 2.2.3, was glued to the sample table with conductive adhesive. Gold was sprayed with Oxford Quorum SC7620 sputter coater (Quorum Technologies, UK) for 45 s (10 mA), and then photographed with ZEISS Sigma 300 SEM (ZEISS Group, Germany) with an accelerated voltage of 3 kV.

#### 2.4.2. XRD analysis

XRD analysis was carried out according to the method proposed by Li et al. (26) with some modifications. The obtained crystals were ground into a fine powder and measured by a D8 Advance X-ray (27) diffraction platform (Bruker Corporation, USA). The scanning speed was 10°/min with a scanning range (2θ) of 5°-50°.

#### 2.4.3. FTIR analysis

FTIR analysis of β-CD, GO, and the prepared IC particles, as mentioned in Section 2.2.3, was performed using a Nicolet 380 spectrometer (Thermo Fisher Scientific, USA). Samples were compressed into thin slices with KBr and scanned in the range of 4000–400 cm^−1^ to obtain the transmittance curves.

#### 2.4.4. Contact angle analysis

The contact angle was measured according to the previous method with some modifications (27). In brief, three-phase contact antennae (oil–water–solid) were measured by the site-drop method using an Attension Theta Flex optical contact angle instrument (Biolin Scientific Co. Ltd., Sweden). ICs powder was made into a relatively smooth sheet with a diameter of 15 mm and a thickness of 1.5 mm under the action of a 10–12 MPa hydraulic press (FW-4, Tianjin Tianguang New Optical Instrument Technology Co., Ltd.). The sheet was then gently put into a rectangular transparent glass pool (25 mm×25 mm×25 mm), in which 5 mL of GO was added in advance. A drop of deionized water (approximately 8 μL) was added to the surface of the sheet, and a high-speed camera was used to take pictures. One Attension software (Biolin Scientific Co. Ltd., Sweden) was used for balance processing to obtain the value of a three-phase contact antenna.

#### 2.4.5. Gas–water surface tension analysis

An Attension Theta Flex optical contact angle instrument (Biolin Scientific Co. Ltd., Sweden) with a CMOS camera available for droplet image processing was used to measure the air–water surface tension of β-CD solution by using the suspended drop method. A trace β-CD aqueous solution was injected into a 200 μL injection pipettor, and 12 μL solution was dripped out within 6 s. The image collection system collected droplet images every 0.2 s. The surface tension was determined by the rapid collection of droplet images, edge detection, and fitting of the Laplacian–Young equation. The entire analysis was conducted at 25°C.

#### 2.4.6. Oil–water interfacial tension analysis

The oil–water interfacial tension of β-CD aqueous solution/GO was measured by using the suspension drop method using the Attension Theta Flex optical contact Angle instrument (Biolin Scientific Co. Ltd., Sweden) with a CMOS camera for dropper image processing (27, 28). The electric injection pipettor was used to absorb the β-CD water solution, and the pipettor tip was then inserted into the transparent rectangular glass dish containing the GO (25 × 25 × 25 mm). The sample was pushed into GO to form a suspension. After 30 s of equilibrium, the rapid collection of the liquid drop image, edge detection, and the fitting of the Laplacian–Young equation were carried out to calculate interfacial tension.

## 3. Results and discussions

### 3.1. Physical properties of emulsion

**Figure 1:**
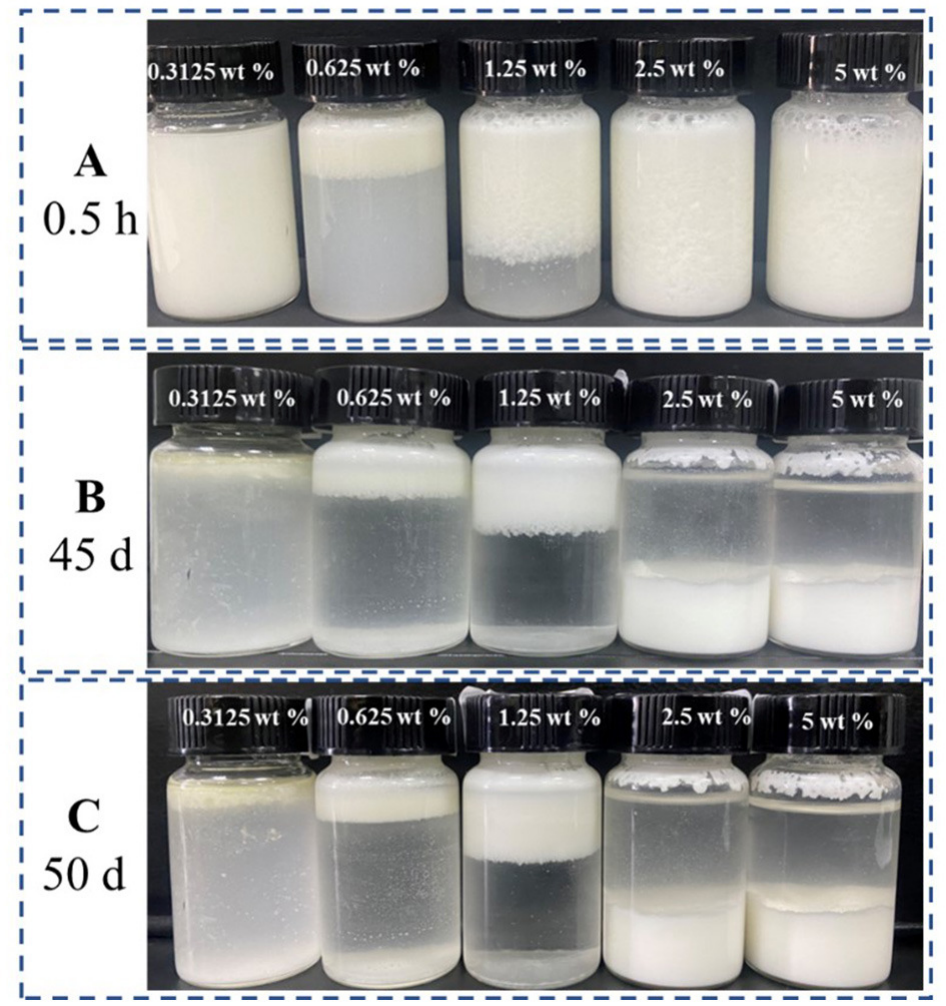
Storage stability of GO/β-CD emulsion **(A)** for 0.5 h, **(B)** for 45 d, and **(C)** for 50 d.

The emulsion stability against coalescence was evaluated by CI, as shown in Figure 1. When GO was added to the β-CD aqueous solution, white flocs gradually appeared at the interface of the two phases and diffused into the water phase. Such phenomenon may be attributed to the host–guest interaction of β-CD and GO to form ICs. The thermal movement of the formed ICs under the action of concentration difference and other driving forces led to spatial migration, and the “glass wool” type structure was formed (29). After high-speed shearing, the two phases became a homogeneous emulsion system. However, such emulsion was unstable, and layering appeared within a short period of time. As shown in Figure 1A, the upper layer was a white “emulsion layer”, while the lower layer was a transparent “clear layer”. With the prolongation of storage time, the emulsions changed obviously. After 45 days of storage, the emulsified layers of the 5 wt% and 2.5 wt% systems settled to the bottom (in this study, the CI value of the emulsions that settled to the bottom was set as a negative value), and the upper layer became clear. Among them, the CI values of the 2.5 wt% and 1.25 wt% systems became smaller, but no demulsification occurred (Figure 1B); while the 0.625 wt% system had oil phase precipitation and floated on the top layer, but a thin emulsion layer was still formed at the phase interface (Figure 1C).

**Figure 2:**
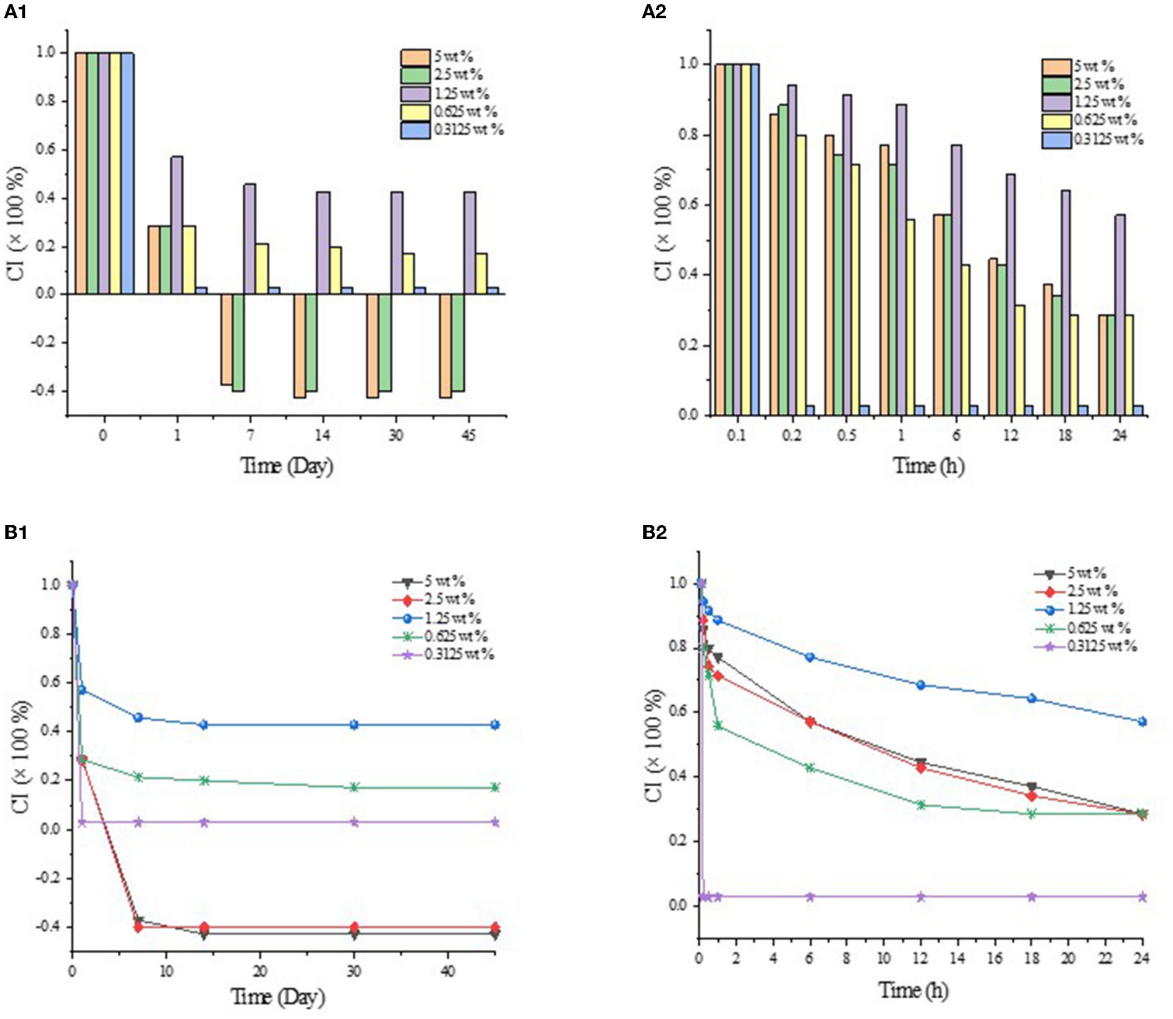
CI values of GO/β-CD emulsion in different storage times. **(A1)** and **(B1)** for CI values at 45 d, **(A2)** and **(B2)** for CI values at 24 h.

As shown in Figure 2, the emulsion formed by GO and β-CD had poor anti-coagulation stability in the short term, and the formed emulsion droplets were relatively large and rough. After long-term storage, when the static time reached the dynamic equilibrium, the CI values no longer changed, and the system tended to be stable. Meanwhile, the emulsion droplets became more delicate. In other words, β-CD microcrystals adsorbed at the oil–water interface to form an adsorption layer or an adsorption film with a certain thickness (2). When two emulsion droplets were close to each other, the adsorption layer overlapped or compressed, which corresponded to the macroscopic behavior of floating or sinking of the emulsion layer during the experiment. In order to maintain dynamic stability, the emulsion itself produced a steric repulsion effect to prevent the emulsion droplets from approaching further. However, in the gravitational field, when the net force on the emulsion drops tended to gravity, the emulsion droplets in the cream layer settled to form a sedimentation layer. At this point, when a centrifugal force was given to the emulsion of the sedimentation layer, part of the sedimentation layer at the bottom became an emulsion layer and float on the top of the system again. While the emulsion with a relatively low concentration of β-CD was demulsified directly (Supplementary Figure S1).

### 3.2. Emulsion type analysis

**Figure 3:**
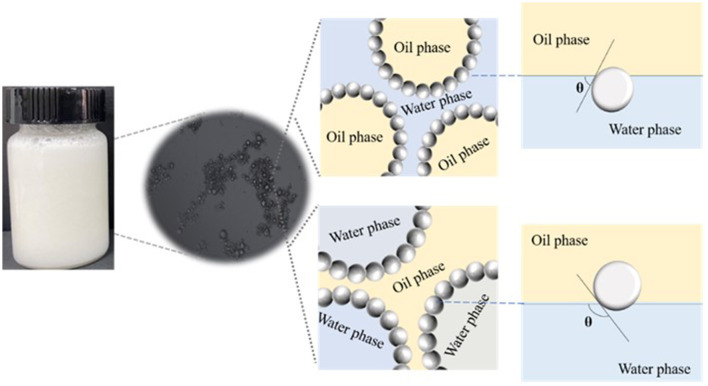
Typical structure of Pickering emulsion.

Solid particles are known to stabilize emulsions by their partial wettability at the oil–water interface, *i.e*., three-phase contact angle (θ). When θ is <90°, the emulsion tends to be O/W type. On the contrary, when θ is larger than 90°, the emulsion tends to be of W/O type. When θ is close to 90°, the particles in the system are most suitable for O/W emulsion with the best stability (Figure 3). To verify the type of emulsion, the θ values of β-CD and ICs microcrystals were characterized. The results showed that the θ value of natural β-CD was almost zero, while for GO/β-CD microcrystals were <90°, which demonstrated that such particles tend to form O/W emulsions (30).

### 3.3. Particle size analysis and microstructure observation

**Figure 4:**
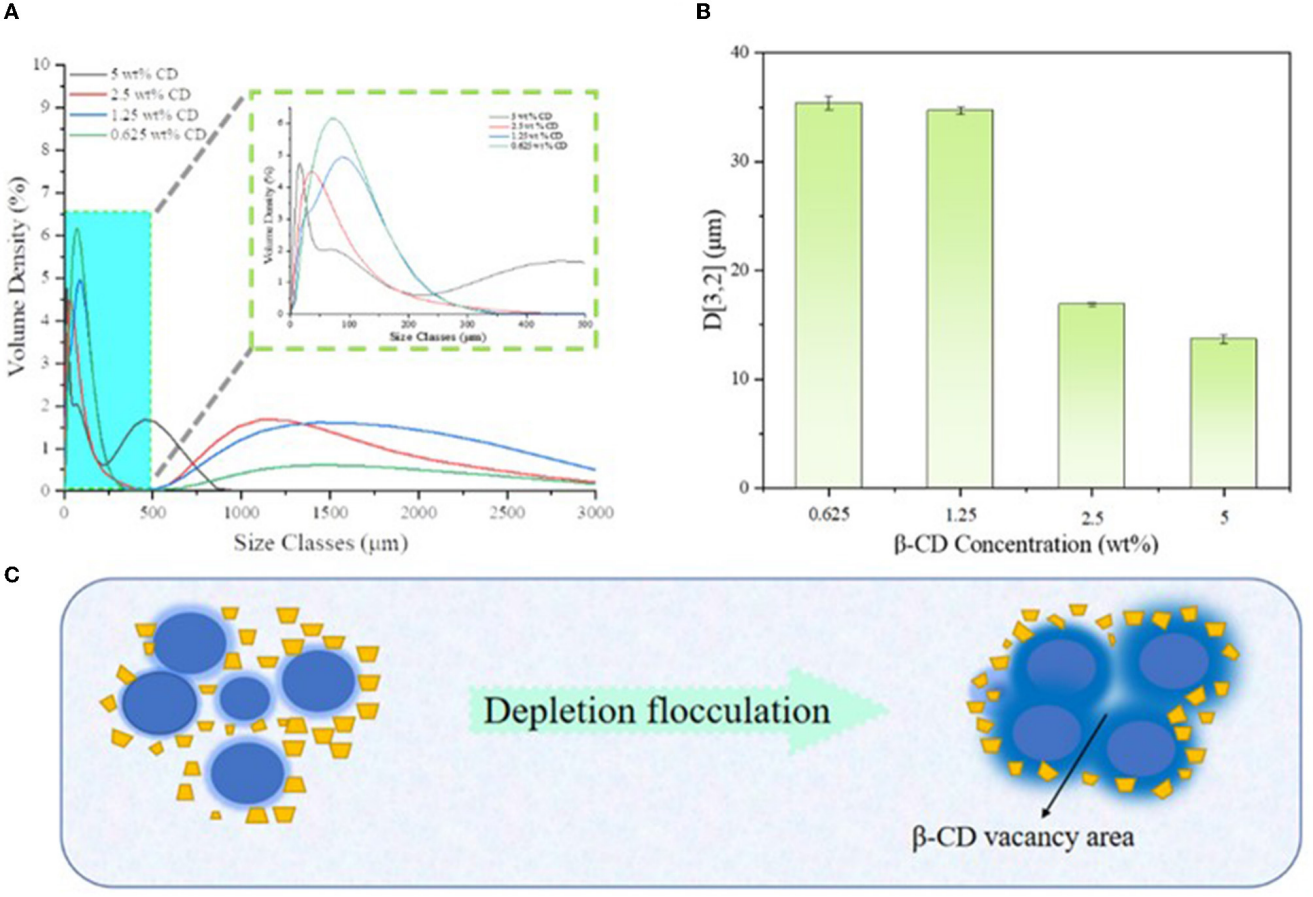
Particle size distribution of different β-CD emulsions **(A)**, corresponding *D*_[3,2]_ values **(B)**, and schematic diagram of emptying flocculation **(C)**.

**Figure 5:**
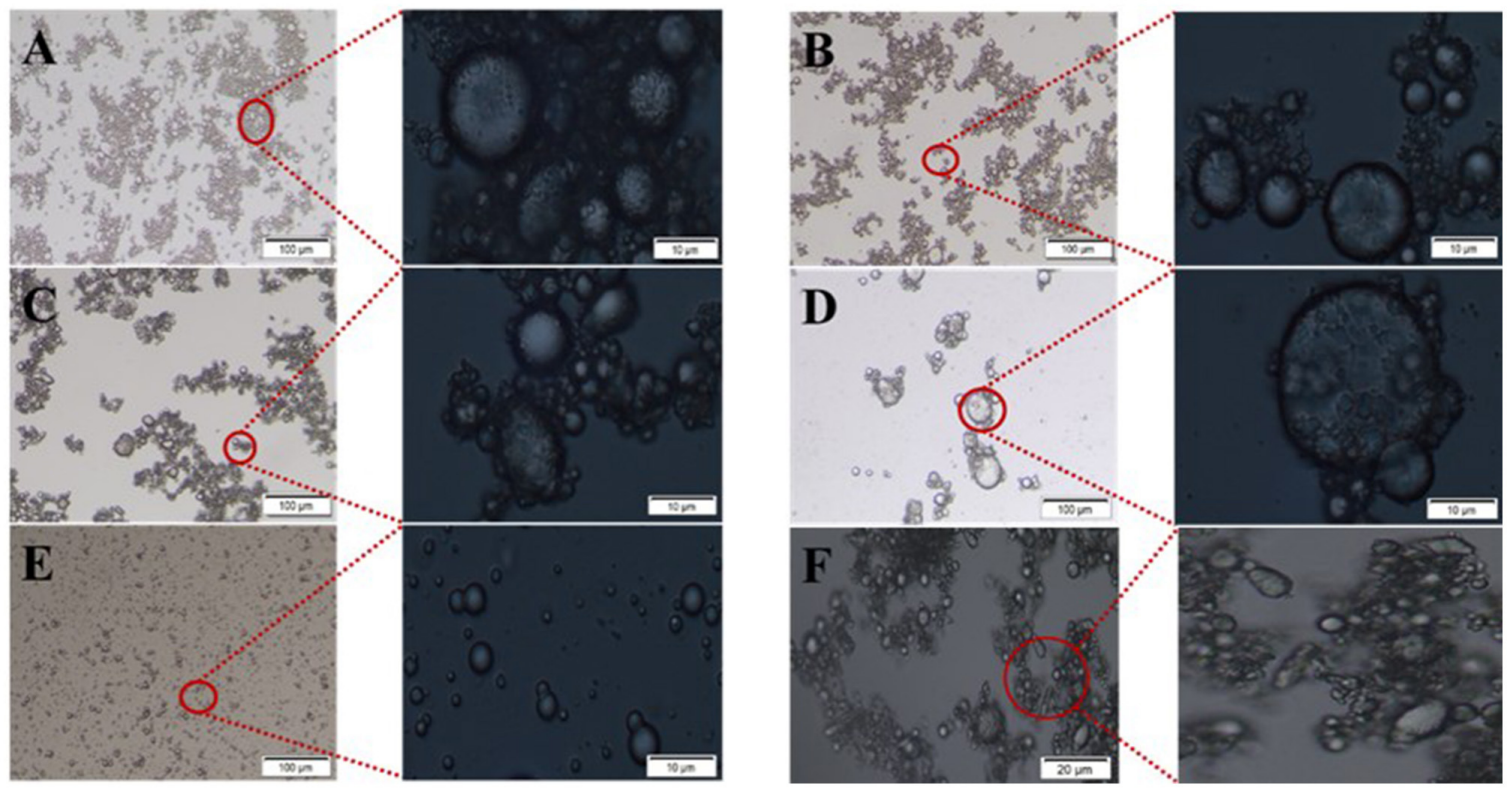
Optical microscope images of Pickering emulsions with different β-CD concentrations. **(A–E)** β-CD of 5 wt%, 2.5 wt%, 1.25 wt%, 0.625 wt%, and 0.3125 wt%; **(F)** was non-spherical droplets in the emulsion with β-CD concentration of 1.25 wt%.

Figure 4 shows the particle size distribution of the emulsion droplets, where the sample with 0.3125 wt% β-CD concentration did not form a stable emulsion, thus, the characterization was abandoned. Obviously, the particle sizes of the samples were mainly concentrated in two intervals, and there were a large number of droplets larger than 20 μm in all emulsion samples (Figure 5), which may be due to the depletion interaction between emulsion droplets. Specifically, larger numbers of hydroxyl groups on the surface of β-CD accelerated its flocculation when diluted with deionized water. During the dilution process, the concentration of β-CD molecules in the space between the emulsion droplets decreased significantly, forming a certain vacancy region, and there was a certain osmotic pressure between the vacancy region and the emulsion phase, which led to the β-CD molecules in the vacancy region tended to flow out of the region, and finally led to the further reduction of the distance between the emulsion droplets and flocculation.

**Figure 6:**
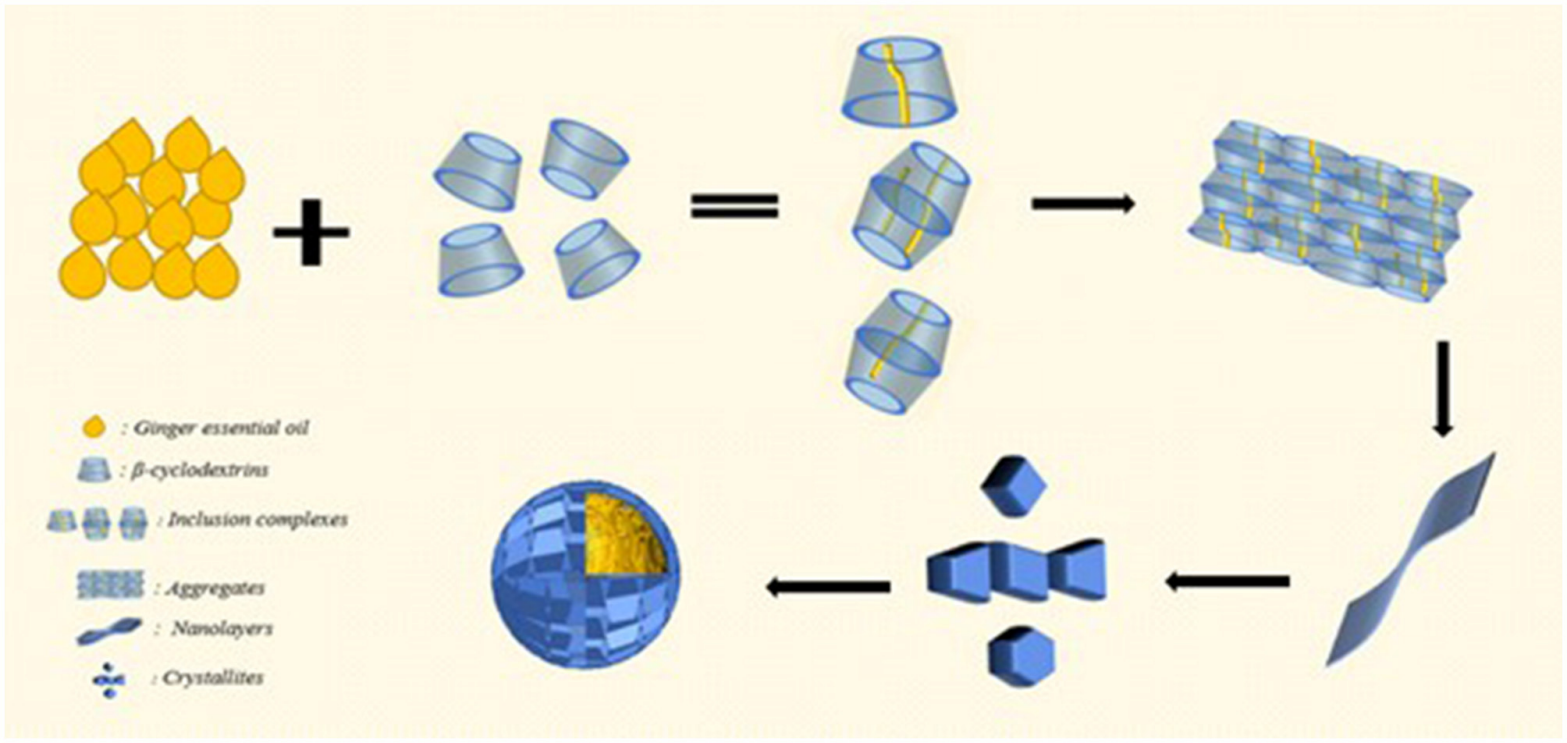
Schematic diagram of β-CD-stabilized emulsion.

The observation of emulsion morphology by optical microscope can directly reflect the internal structure information of the emulsion droplets, and the agglomeration phenomenon between emulsion droplets could be easily observed, as shown in Figure 4. In the undiluted condition, the droplets were agglomerated due to the intermolecular cohesion of oil and the hydrogen bonding of β-CD, and a large number of flocs were observed around the droplets. When the β-CD was in excess, the -OH in the non-emulsified β-CD was further linked to the adjacent β-CD through hydrogen bonding to form nanoparticles, which subsequently caused self-agglomeration. In addition, the instability of the interfacial film caused by excessive shearing may also cause aggregation. When the β-CD content in the emulsion was 1.25 wt%, some emulsion droplets were non-spherical (Figure 5F). This phenomenon was called “interfacial jamming” (31, 32), which was caused by the high packing density of the adsorbed β-CD particles at the oil–water interface. The excessively high packing density made the particles lose their freedom due to the limited volume, and the interfacial film structure changed from a liquid-like free-flowing state to a solid-like state, and a blocking phase transition occurred in the interfacial film (33). Studies have found that interfacial jamming can be regulated by adjusting the type, volume fraction, and interaction force of colloidal particles, thereby realizing the phase transition of emulsions (29, 34). In the enlarged image (Figure 6), it can be found that the surface of the emulsion droplets was rough, and some regular rectangular and rhomboid sheets appeared on the interface under a specific concentration. Therefore, it can be speculated that these regular lamellar crystals (large particles) were amphiphilic lamellar inclusions formed by further self-assembly of the supramolecular (small particles) formed by β-CD and GO. Similarly, Ishimoto et al. (35) also confirmed the formation of crystalline flake shells between host and guest in the study of the formation of γ-CD and cholesterol oleate in drug-carrying nanocarriers.

### 3.4. Emulsion interface structure analysis

**Figure 7:**
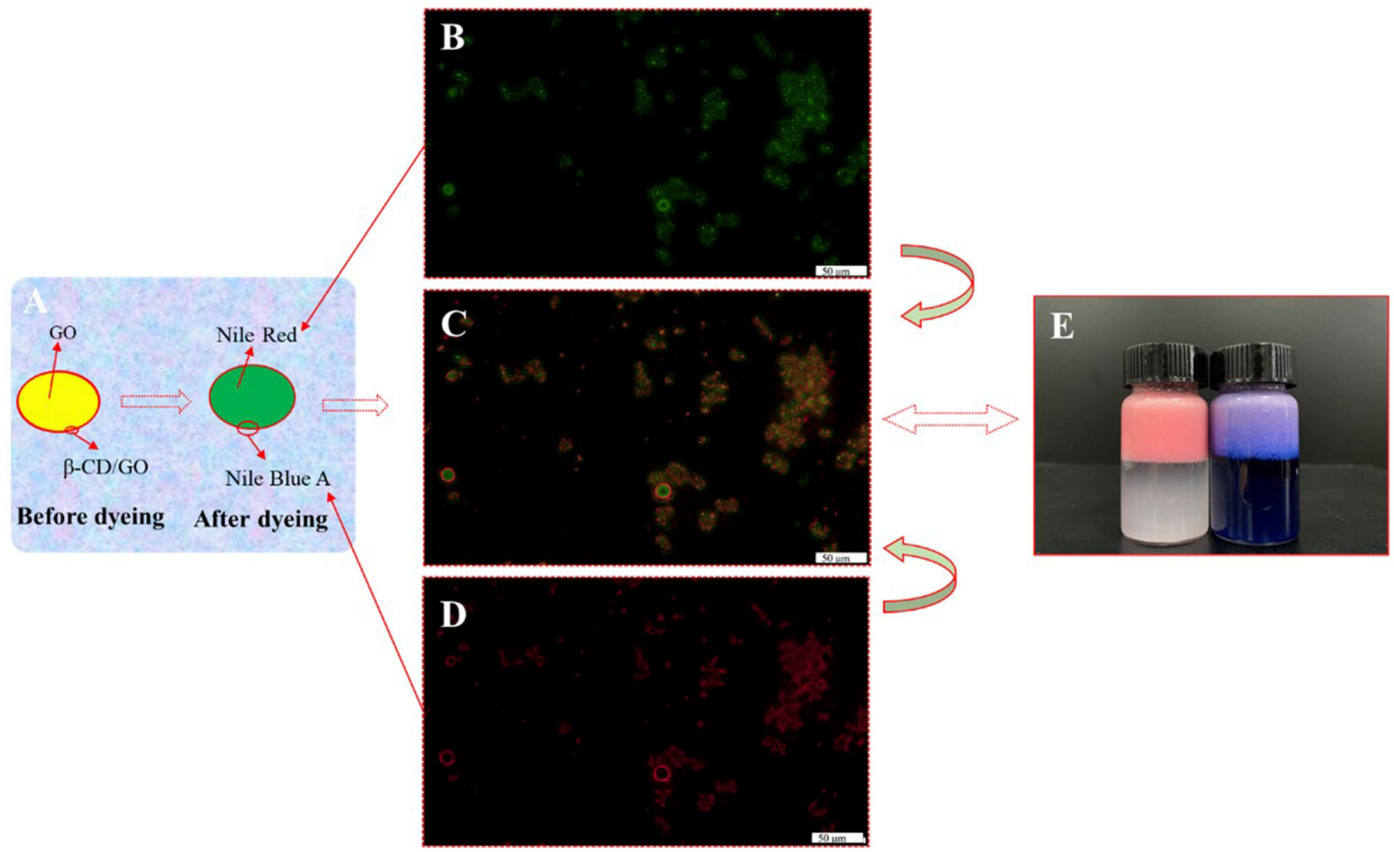
Fluorescence micrographs of GO/β-CD (1.25 wt%) Pickering emulsion. **(A)** The control model before and after staining; **(B)** was GO; **(C)** was the composite fluorescence image; **(D)** was the β-CD; **(E)** was the dyed emulsion after storage for 30 days (the left bottle was only dyed GO phase, and the right bottle was dyed both two phases).

In order to better characterize the distribution of particle emulsifiers in Pickering emulsion formed by β-CD and GO, the fluorescence microscopic observation on the emulsion with the concentration of β-CD 1.25 wt% was carried out, as shown in Figures 7A–E. The fluorescence-stained GO and β-CD showed green (Figure 7B) and red (Figure 7D), respectively, under the irradiation of the corresponding wavelength excitation light. The analysis software of the instrument combined the two photos to get Figure 7C, and it was easy to see that most of the red fluorescence-labeled β-CD aggregated into a semi-dense film on the surface of the green fluorescence-labeled GO droplets, forming a red–green “sandwich” structure, which reflected the fact that the particle emulsifier adsorbed on the oil–water interface to form O/W Pickering emulsion. In addition, the dispersion of the droplets was poor, and the β-CD particles were unevenly distributed and seriously agglomerated at the interface by observing the surface structure of a single droplet. These results indicated that the emulsification ability of the particles formed by β-CD and GO was relatively weak, which was an important reason for the short-term instability of the formed emulsion (36).

### 3.5. Interface self-assembly mechanism of particles formed by β-CD and GO

**Figure 8:**
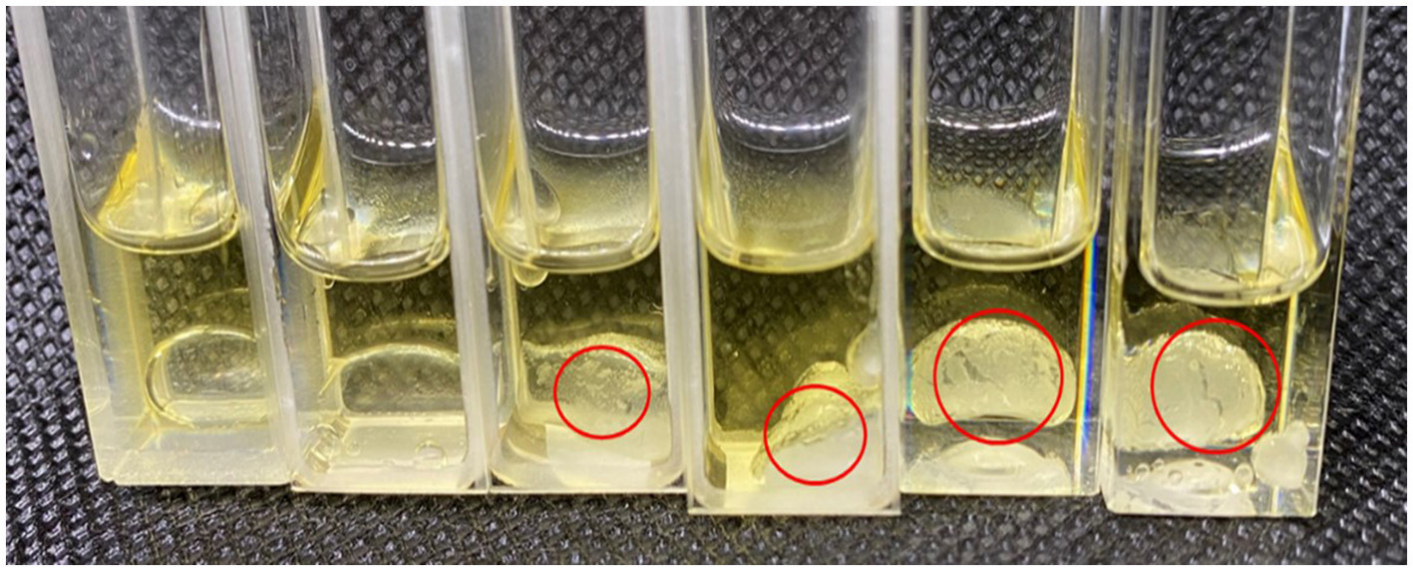
Formation of interface film (From left to right: 0.2 mL deionized water, 0.3125 wt%, 0.625 wt%, 1.25 wt%, 2.5 wt%, and 5 wt% of β-CD solution were added to 1 mL GO).

Based on the analysis of the physical and chemical properties of the emulsion, it was speculated that the interfacial self-assembly of the supramolecular emulsion formed by β-CD and GO constructed highly ordered ultra-high molecular aggregates (large particles) under the balance of various forces, and these aggregates were the building blocks for further self-assembly. When amphiphilic supramolecules were used as building blocks, their self-assembly behavior determined the formation of Pickering emulsion systems. In the experiment, the transient process changes of β-CD at the oil–water interface were captured during measuring the surface tension by using the suspension drop method, as shown in Supplementary Video 1. In addition, to more intuitively observe the small IC particles formed by β-CD between the two phases, the deionized water and β-CD solution were added to GO, respectively. As shown in Figure 8 and Supplementary Figure S2, the interface film formed by β-CD and GO became clearer as the concentration increased.

#### 3.5.1. Molecular states of GO/β-CD ICs

**Figure 9:**
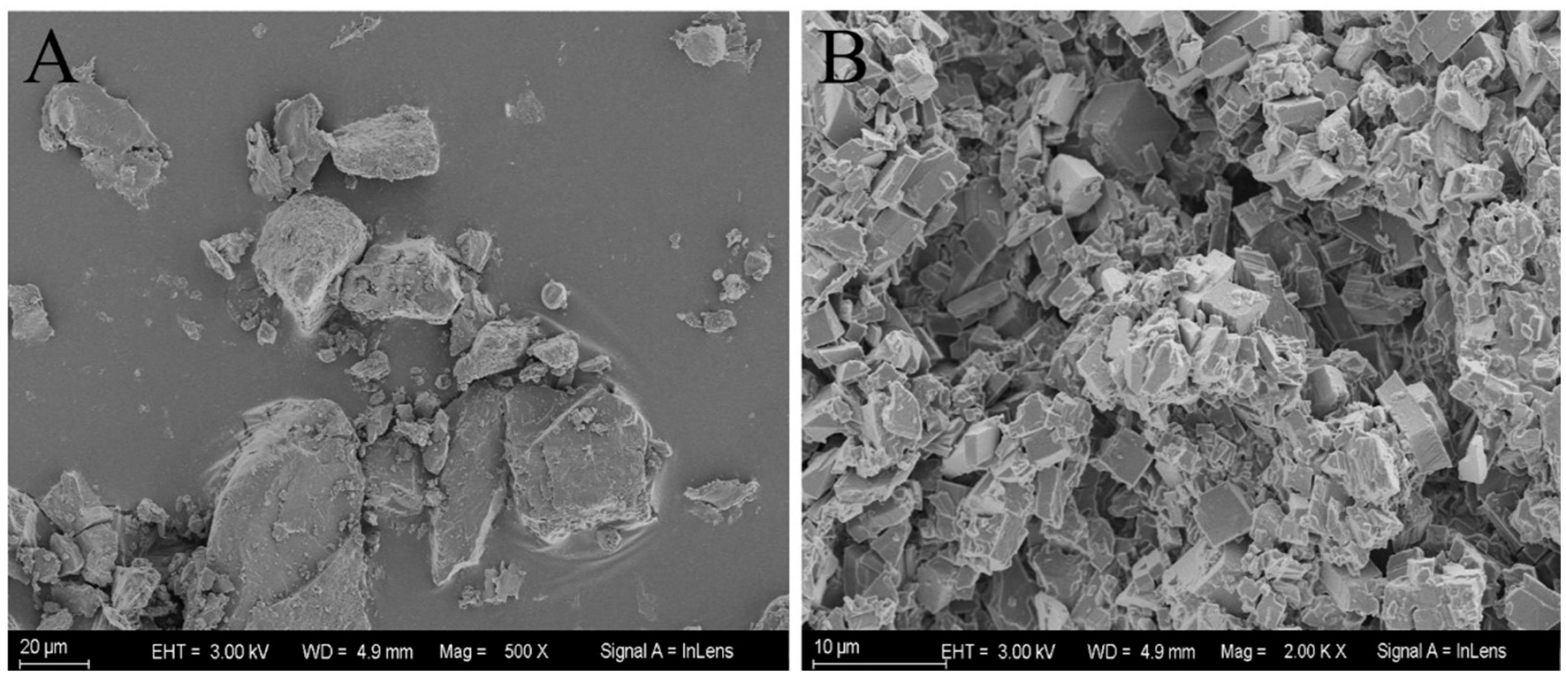
SEM images of β-CD **(A)** and GO/β-CD IC particles **(B)**.

The morphology of IC particles was characterized by using an SEM. As shown in Figures 9A, B, β-CD presented blocky particles with large particle sizes; on the contrary, GO/β-CD particles were small with a specific cubic shape and flat surface. The host–guest interaction between β-CD and GO made GO weaken the hydrogen bond of β-CD during the inclusion process, forming a supramolecular structure (small particles), and then the supramolecular gradually self-assembled to form a layered structure. The layered structure is stacked to form regular large particles with a certain thickness. This result was consistent with the surface structure observed by microscopy in Figure 5. In addition, the TEM results of the interaction between β-CD and 1-decanol reported by Pacaud et al. (37) also found a similar phenomenon, who believed that the flaky crystals were formed by the gradual development of IC particles.

**Figure 10:**
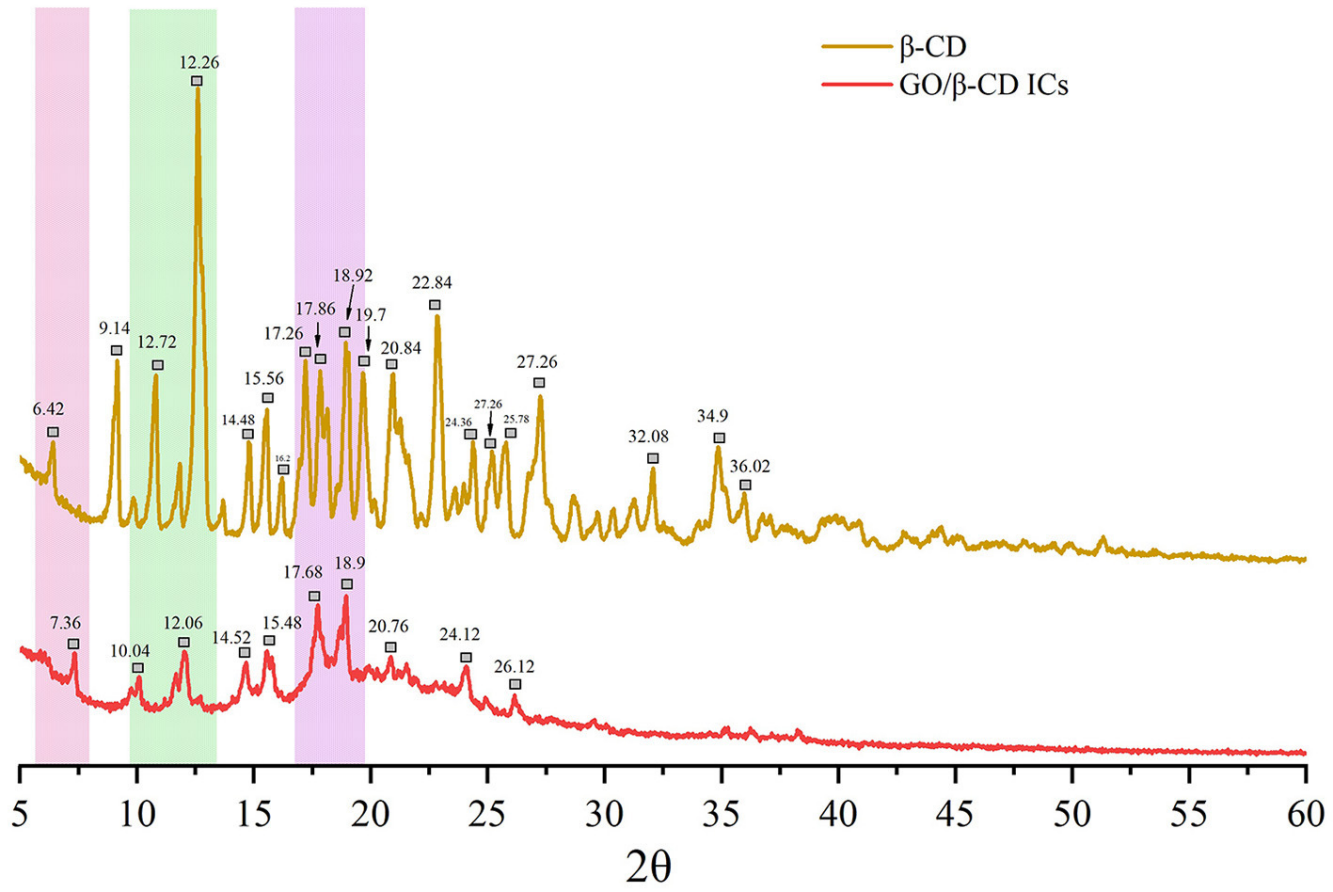
XRD patterns of GO/β-CD ICs and β-CD powder.

The molecular state of GO/β-CD IC particles was evaluated by XRD in the range of 5°-60°. As shown in Figure 10, β-CD was a typical crystal structure with many strong crystal absorption peaks. It was not difficult to find in Figure 10 that the XRD pattern of the GO/β-CD had some important changes, and the absorption peaks at 7.3°, 10.04°, and 12.06° were consistent with the absorption peaks of β-CD, while the absorption peak of GO/β-CD ICs shifted. Compared with β-CD, ICs had no strong absorption peaks at 9.14° and 30–60°. In addition, the ICs had different absorption peaks from β-CD at 14.52°, 15.48°, 20.76°, 24.12°, and 26.12°. The aforementioned changes can be attributed to the interaction between host and guest components, which ultimately affected the molecular structure of β-CD (38). Both the appearance of new peaks and the disappearance of characteristic peaks proved the formation of ICs and results strongly confirmed that β-CD and GO self-assembled at the oil–water interface to form GO/β-CD ICs with a crystal structure and partial crystallinity. Similar results were also confirmed by Li et al. (26). Notably, the formation of host–guest IC crystallites further illustrated that the self-assembly of β-CD at the oil–water interface was a different O/W emulsion stabilization mechanism, which began at the adsorption of ICs formed by β-CD and guest at the oil–water interface, followed by the gradual formation of microcrystals anchored on the phase interface, finally formed a Pickering emulsion.

#### 3.5.2. FTIR analysis of GO, β-CD, and ICs

**Figure 11:**
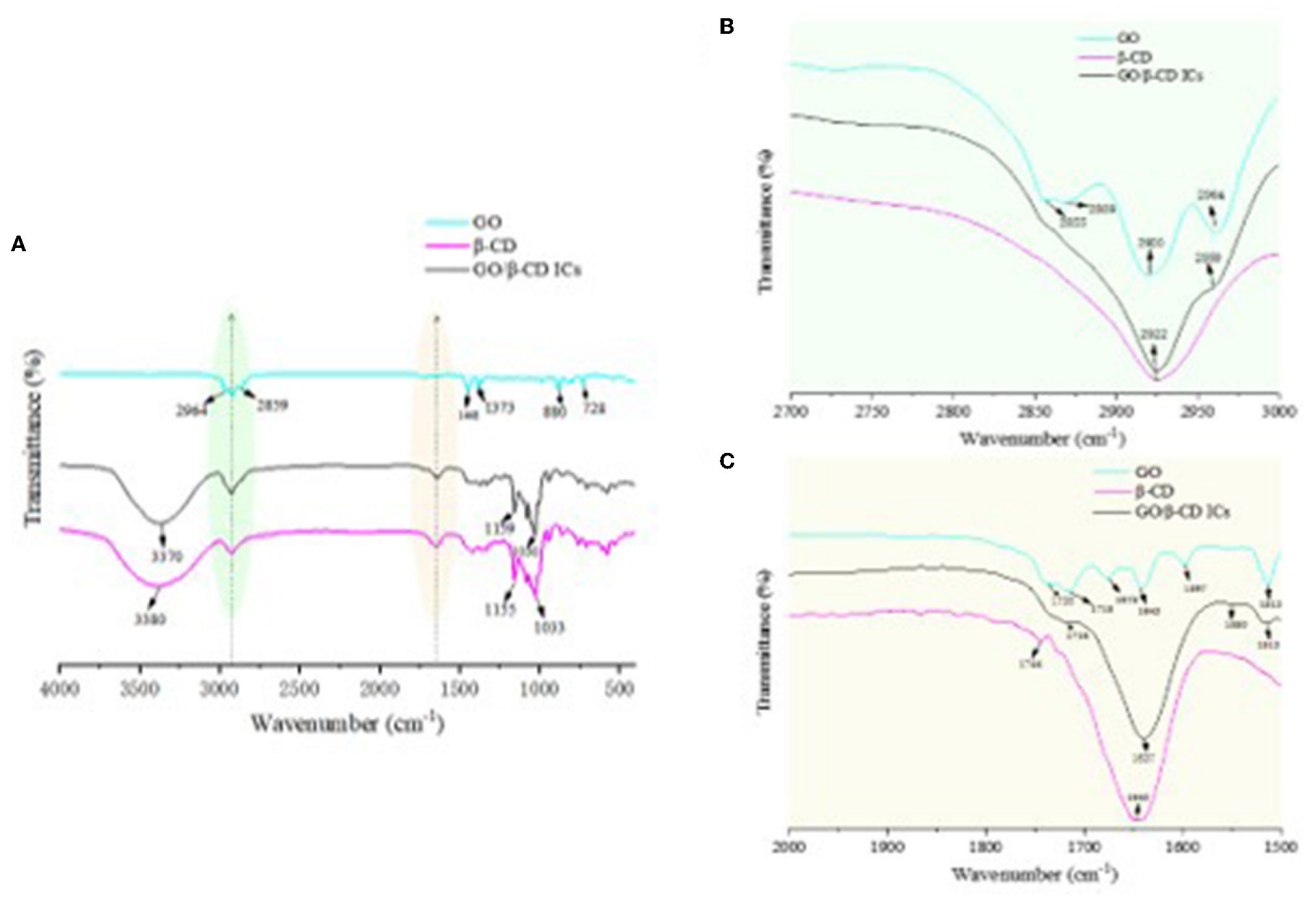
FTIR spectrum of β-CD, GO/β-CD ICs, and GO **(A–C)** were the local magnification of the green and yellow shadows, respectively.

In order to prove that there was no chemical reaction between β-CD and GO, FTIR characterization of β-CD, GO, and their ICs was carried out. As can be seen in Figure 11, GO contained -CHO groups, -COOR groups, and benzene rings. The absorption peaks near 1640 cm^−1^ were C-H stretching vibrations, and the absorption peaks at 2,964 cm^−1^ and 2,855 cm^−1^ belonged to the C-H antisymmetric stretching vibration in methylene, and the C-H stretching vibration peak in methoxy (19). The absorption peaks at 728, 1373, and 880 cm^−1^ were the stretching vibrations of the benzene ring and C=O and C-H on the benzene ring, respectively (21). In the FTIR spectrum of β-CD, -OH appeared at the wave number of 3380 cm^−1^. The stretching vibration peak of 2922 cm^−1^ belonged to the C-H stretching vibration (Figure 11C) (32). Due to the intermolecular hydrogen bond association, the benzene ring in GO entered the hydrophobic cavity of β-CD through hydrophobic interaction, resulting in the disappearance or weakening of the absorption peaks of the stretching vibration bonds of C-C and C-H on the benzene ring (Figure 11B) (37). The absorption peak of -OH vibrational stretching in GO/β-CD ICs had a blue shift phenomenon, which deviated from the wavenumber of 3,380 cm^−1^ to 3,370 cm^−1^. Overall, the FTIR spectrum of ICs and β-CD were similar, and there were no new chemical bond absorption vibration peaks appeared in ICs, which further confirmed the conclusion that GO was successfully encapsulated in the β-CD cavity through host–guest interaction and the formed ICs exhibited partial crystallinity of β-CD.

#### 3.5.3. Influence of β-CD on the surface and interfacial tension

**Figure 12:**
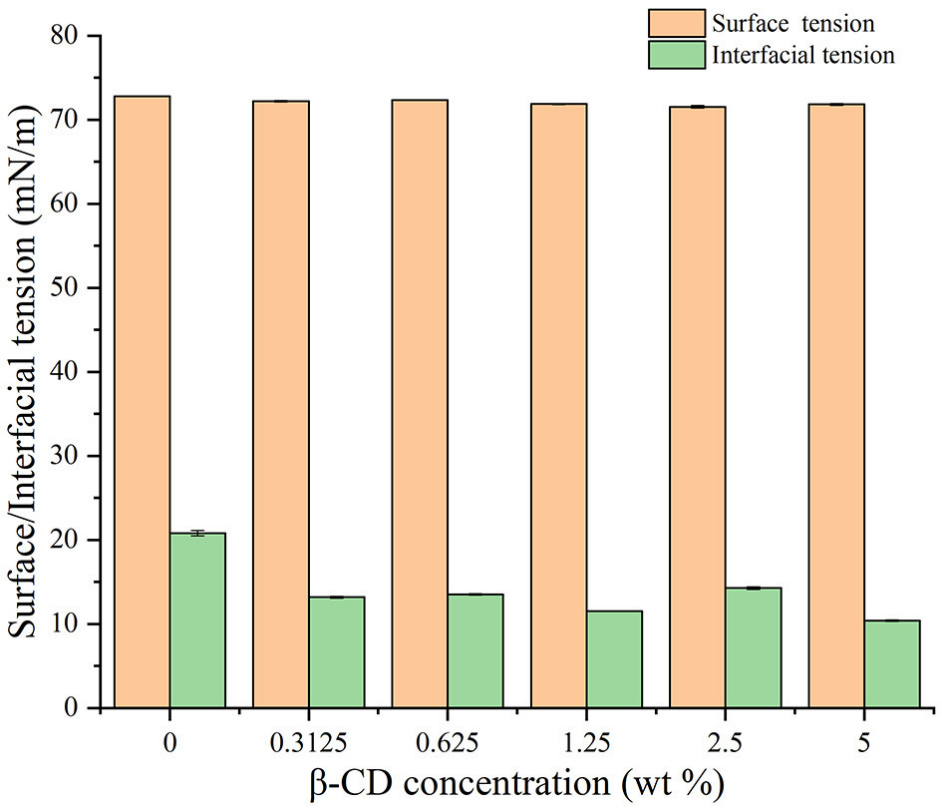
Effects of different concentrations of β-CD on water–vapor surface tension and oil–water interfacial tension.

The values of surface and interfacial tension were measured by means of the Young–Laplace equation fitting using the suspension drop method. As shown in Figure 12, the surface tension of water at 25°C was 72.8 mN/m, and the addition of β-CD has little effect on the surface tension of water. On the contrary, the interfacial tension between oil and water at 25°C was approximately 20.8 mN/m, and with the increase in β-CD concentration, the interfacial tension first gradually decreased and then increased. When the concentration was too high (5 wt%), β-CD formed a rigid shell due to interfacial jamming, which made the interfacial tension of oil and water became very low (approximately 10.4 mN/m). Due to this, the emulsion formed under such concentrations also became unstable (26, 39, 40). When β-CD was 1.25 wt%, the interfacial tension between oil and water had a good value of 11.52 mN/m, and the emulsion was also in a good state. In conclusion, natural β-CD had no surface activity on the water/gas surface (39). However, β-CD was soluble in water, and the hydrophobic parts of guest molecules could be combined into its hydrophobic cavity, forming ICs. The ICs of GO and β-CD had a traditional surfactant structure, in which β-CD was a hydrophilic head, and the molecular chain of GO connected to β-CD and exposed outside was a hydrophobic tail. Such structure is self-assembled at the oil–water interface, which, in turn, lowered the oil–water interfacial tension. Previous studies had concluded similar results (39, 40).

#### 3.5.4. Three-phase contact angle (θ) of β-CD and GO/β-CD ICs

**Figure 13:**
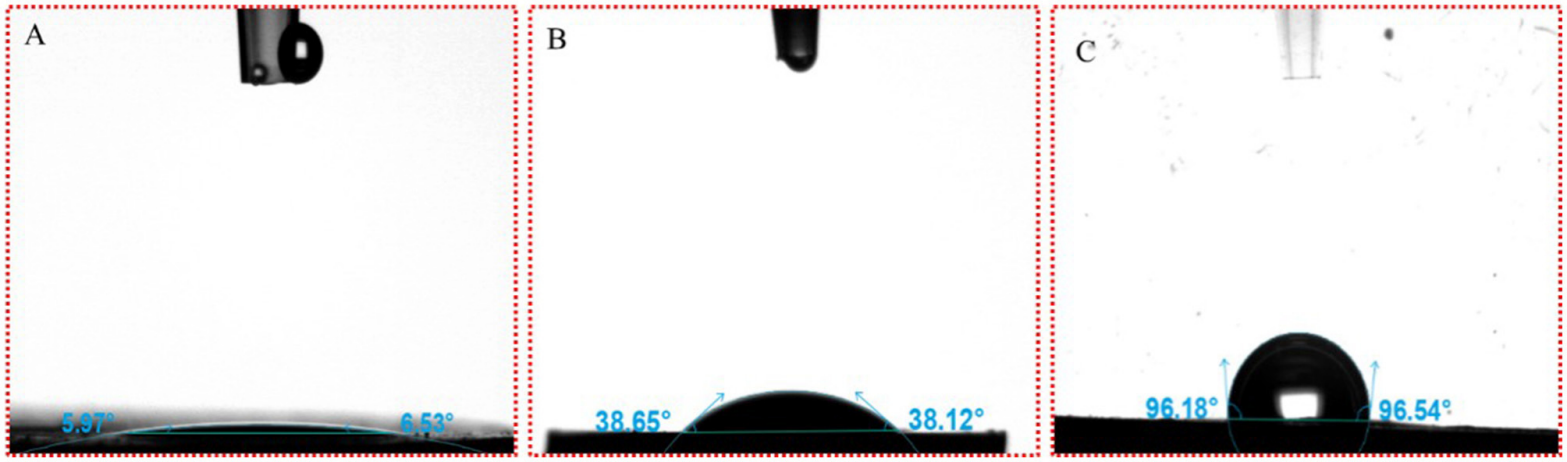
Three-phase antennae (θ) images of water droplets on β-CD and GO/β-CD ICs. **(A, B)** β-CD and GO/β-CD ICs at the water–air interface; **(C)** was for water–oil interface of β-CD.

The wettability of small and large particles formed by β-CD and GO was characterized by testing the three-phase contact angles (θ) formed by these particles with the oil–water interface (27). The θ of water droplets on β-CD pellets in the air was within 10° (Figure 13A), indicating that β-CD was highly hydrophilic, which was related to the ability of β-CD to dissolve in water. The θ of prepared GO/β-CD ICs microcrystals, as mentioned in Section 2.2.2, was 38.65° (for the left side) and 38.12° (for the right side), as shown in Figure 13B, which were larger than that of β-CD, indicating that β-CD and GO formed ICs microcrystals with enhanced lipophilicity. Finally, since it was difficult to determine the θ of β-CD with the small particles formed by GO (the particles in the red circle in Figure 8), the measurement of β-CD substrate in the oil–water interphase was carried out. As shown in Figure 13C. After equilibrating for 30 s, the left θ of the water droplet was 96.18°, and the right θ was 96.53°. In general, when 60° < θ < 120°, the desorption free energy of the particles was higher, which could form an ultra-stable Pickering emulsion. In fact, the stability of the emulsions we prepared was not consistent with it, which may be related to its low interfacial tension (27). In conclusion, the results of θ confirmed from a more professional aspect that the small particles and the large particles of ICs formed by GO and β-CD self-assembly at the interface were amphiphilic, and they all participated in the formation of O/W Pickering emulsion.

## 4. Conclusion

In the present study, O/W Pickering emulsion could be formed by β-CD and GO without an additional emulsifier. β-CD had process changes during the formation of the emulsion, and this process had continuity and instantaneity. The surface of the emulsion droplets was rough with some lamellar particles attached to the surface of the droplets. FTIR, XRD, and SEM analyses confirmed that these particles were ICs formed by the interaction between β-CD and GO. During the formation of the Pickering emulsion, β-CD and GO first formed the amphiphilic ICs through the interaction of host and guest, and then the ICs further self-assembled to form the amphiphilic ICs microcrystals; the crystal particles aggregated at the oil–water phase interface, forming Pickering emulsion, and the aforementioned three processes existed simultaneously.

The analysis of the self-assembly of the ICs formed by β-CD and GO had important guiding significance for the construction of CDs supramolecular Pickering emulsion. However, we only partially studied the discovered phenomenon, and further exploration is needed on how the lamellar parent crystals are formed and what monomers in the GO interacted with CDs. There is no doubt that the study of these problems is challenging, but the solution to these problems will promote the further development of CD Pickering emulsions.

## Data availability statement

The raw data supporting the conclusions of this article will be made available by the authors, without undue reservation.

## Author contributions

XK and XZ: conceptualization, methodology, project administration, data curation, and writing—reviewing and editing. QK and QM: methodology, writing—reviewing and editing, and supervision. All authors contributed to the article and approved the submitted version.

## Supplementary material

Supplementary Video 1 Transient process changes of β-CD at the oil–water interface.
