# Supplementary material for: Pickering emulsions stabilized by β-CD microcrystals: Construction and interfacial assembly mechanism
Source: Front Nutr. 2023 Mar 22;10:1161232. doi: 10.3389/fnut.2023.1161232 (PMC10073450; doi:10.3389/fnut.2023.1161232)
Supplement: Supplementary file 2 [file Data_Sheet_1.PDF]

Supplementary Material

Pickering emulsions stabilized by β-CD microcrystals: Construction and interfacial assembly mechanism

Xingran Kou, Xinping Zhang, Qinfei Ke*, Qingran Meng*

*** Correspondence:** Qinfei Ke: kqf@sit.edu.cn and Qingran Meng: qmeng@sit.edu.cn

1 Supplementary Figures

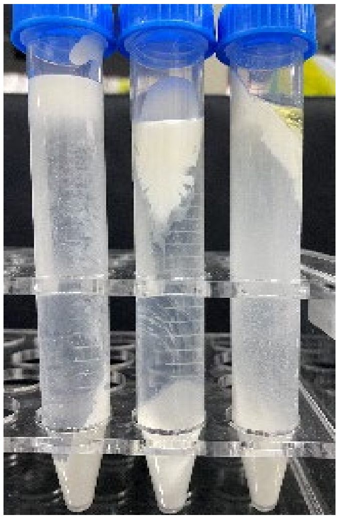

Supplementary Figure 1. After centrifugation at 10000 rpm for 5 min, the precipitation was washed with deionized water for the second time. From left to right were 5 wt %, 2.5 wt %, 1.25 wt % β-CD stabilized emulsion. Among them, 5 wt% and 2.5 wt% were divided into three layers (upper emulsion layer, middle water layer, and lower precipitation layer), and 1.25wt % was divided into four layers (upper oil phase, upper emulsion layer, middle water layer, and lower precipitation layer).

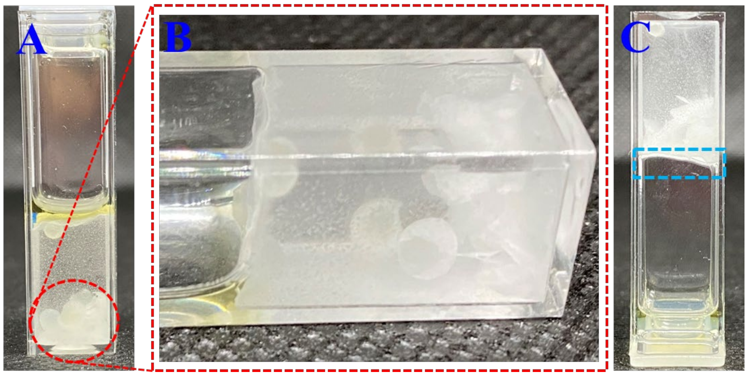

Supplementary Figure 2. The state of the interface layer after GO was added to β-CD aqueous solution for 7 d (A); B was the formed spherical interface film after oil droplet leakage; C was the inverted diagram of A.
